# Differential alteration of fMRI signal variability in the ascending trigeminal somatosensory and pain modulatory pathways in migraine

**DOI:** 10.1186/s10194-020-01210-6

**Published:** 2021-01-07

**Authors:** Manyoel Lim, Hassan Jassar, Dajung J. Kim, Thiago D. Nascimento, Alexandre F. DaSilva

**Affiliations:** 1 Headache and Orofacial Pain Effort (H.O.P.E.), Department of Biologic and Materials Sciences & Prosthodontics, University of Michigan School of Dentistry, 1011 N. University Ave, Room 1014A, Ann Arbor, MI 48109-1078 USA; 2 Michigan Neuroscience Institute, University of Michigan, Ann Arbor, MI 48109 USA

**Keywords:** fMRI, Resting-state, Brain signal variability, Dynamic functional connectivity, Migraine, Pain

## Abstract

**Background:**

The moment-to-moment variability of resting-state brain activity has been suggested to play an active role in chronic pain. Here, we investigated the regional blood-oxygen-level-dependent signal variability (BOLD_SV_) and inter-regional dynamic functional connectivity (dFC) in the interictal phase of migraine and its relationship with the attack severity.

**Methods:**

We acquired resting-state functional magnetic resonance imaging from 20 migraine patients and 26 healthy controls (HC). We calculated the standard deviation (SD) of the BOLD time-series at each voxel as a measure of the BOLD signal variability (BOLD_SV_) and performed a whole-brain voxel-wise group comparison. The brain regions showing significant group differences in BOLD_SV_ were used to define the regions of interest (ROIs). The SD and mean of the dynamic conditional correlation between those ROIs were calculated to measure the variability and strength of the dFC. Furthermore, patients’ experimental pain thresholds and headache pain area/intensity levels during the migraine ictal-phase were assessed for clinical correlations.

**Results:**

We found that migraineurs, compared to HCs, displayed greater BOLD_SV_ in the ascending trigeminal spinal-thalamo-cortical pathways, including the spinal trigeminal nucleus, pulvinar/ventral posteromedial (VPM) nuclei of the thalamus, primary somatosensory cortex (S1), and posterior insula. Conversely, migraine patients exhibited lower BOLD_SV_ in the top-down modulatory pathways, including the dorsolateral prefrontal (dlPFC) and inferior parietal (IPC) cortices compared to HCs. Importantly, abnormal interictal BOLD_SV_ in the ascending trigeminal spinal-thalamo-cortical and frontoparietal pathways were associated with the patient’s headache severity and thermal pain sensitivity during the migraine attack. Migraineurs also had significantly lower variability and greater strength of dFC within the thalamo-cortical pathway (VPM-S1) than HCs. In contrast, migraine patients showed greater variability and lower strength of dFC within the frontoparietal pathway (dlPFC-IPC).

**Conclusions:**

Migraine is associated with alterations in temporal signal variability in the ascending trigeminal somatosensory and top-down modulatory pathways, which may explain migraine-related pain and allodynia. Contrasting patterns of time-varying connectivity within the thalamo-cortical and frontoparietal pathways could be linked to abnormal network integrity and instability for pain transmission and modulation.

## Background

Migraine is a debilitating neurological disorder characterized by recurrent headaches episodes, often accompanied by amplified perception of multiple sensory inputs such as cutaneous allodynia, photophobia, and phonophobia [1–3]. The suggested mechanism in migraine is likely through sensitized trigeminovascular and dysfunctional pain modulatory systems [4–6]. Unlike other chronic pain disorders, migraine has a cycle divided into different stages, including peri-ictal (premonitory, preictal, ictal, and postictal) and interictal period [1]. Thus, it is essential to understand what functional brain abnormalities are present at each stage and how they are associated with migraine attack severity. Resting-state functional magnetic resonance imaging (fMRI) studies employing amplitude of low-frequency fluctuations (ALFF) [7] or conventional static functional connectivity have shown abnormalities in spontaneous brain activity even during the interictal period [8–11].

Moment-to-moment brain signal variability in fMRI resting-state, once regarded as just a noise and thus ignored in the neuroimaging field, has recently been proposed as an indicator of the brain function and its response to an environmental challenge [12–14]. Additionally, it might be an important index for brain function related to pain perception and modulation. In a resting-state fMRI study, healthy subjects with high blood-oxygen-level-dependent signal variability (BOLD_SV_) had low pain sensitivity and better coping ability [15]. In contrast, patients with chronic pain showed heightened BOLD_SV_ in the ascending pain pathway and default mode network, and these abnormalities were related to pain symptoms [16, 17]. Few studies measuring regional brain activity with the ALFF method reported that interictal migraine patients had increased ALFF in the thalamus [8, 10] and decreased in the rostral anterior cingulate cortex and medial prefrontal cortex [10], indicating a disrupted low-frequency oscillation in spontaneous brain activity.

Migraine in the interictal period also exhibited altered static functional connectivity in brain regions associated with nociceptive/antinociceptive processing [9, 11, 18–21] as well as functional networks such as dorsal attention and executive control network [22, 23]. This functional connectivity analysis typically assumes that functional coupling between brain regions is constant across time. In contrast, dynamic functional connectivity (dFC), which considers temporal variations of functional connectivity, has provided novel insight into the understanding of dynamic properties of brain network for acute and chronic pain [16, 24, 25]. Recently, it has been reported that the variability of dFC within the salience network was higher in migraine with aura compared to healthy controls (HC) and migraineurs without aura [26]. Migraine patients exhibit abnormal thalamo-cortical dynamic functional network connectivity between the posterior thalamus and default mode and visual regions [27]. However, the temporal dynamics of brain activity and neural communication during the interictal period and its clinical significance for migraine attacks have not been well studied. To the best of our knowledge, no previous studies have assessed temporal BOLD_SV_ in migraine patients.

In our study, we assumed that the resting-state BOLD_SV_ and dFC could be a useful measure to reveal altered cortical excitability and dysfunctional network dynamics that impact migraine attack and pain. To this end, we examined differences of resting-state BOLD_SV_, defined as the standard deviation of the BOLD signal, in interictal migraine patients compared to the HC group. Compared to the ALFF, calculated as the square root of the power within a specific frequency range, the standard deviation is a direct index of BOLD signal fluctuation in the time domain [13]. We hypothesized that migraineurs would show abnormal BOLD_SV_ in the ascending trigeminal somatosensory and the descending pain modulatory pathways compared to the HC group, and that abnormal BOLD_SV_ would be associated with clinical and experimental pain during the migraine attack. Clinical and experimental pain, including migraine attack area/intensity (and their summation) and thermal pain threshold on the ophthalmic trigeminal region, were assessed during the patients’ ictal phase. In addition, we utilized dynamic conditional correlation (DCC) [28], a reliable method for measuring dFC to examine the temporal dynamics of functional connectivity within the ascending trigeminal somatosensory and pain modulatory pathways.

## Methods

### Study participants

Twenty migraine (episodic or chronic) patients were recruited by local advertisement. Eligibility criteria for migraine patients were: (1) diagnosis as per the International Headache Society Classification (ICHD-3-beta) [29], (2) between 18 and 45 years of age, and (3) willing to stop taking abortive medications within 48 h before the scan. Patients were excluded if they had: (1) opioid or hormonal contraceptive use 6 months before enrollment, (2) other chronic pain disorders, (3) clinically relevant systemic medical and psychiatric illnesses, (4) pregnancy, (5) preventive medication or (6) contraindications for magnetic resonance imaging (MRI) (ie, any metallic devices, pacemakers, metallic implants, or metallic objects in the body) assessments. Age and sex-matched 26 HC subjects were included. Exclusion criteria for HC were the same as for migraine patients, with the exception of the migraine diagnosis. Thus, a total of 20 patients (13 episodic migraine (EM) and 7 chronic migraine (CM)) and 26 HC subjects were included for resting-state fMRI scans **(**Table 1). None of the enrolled migraine patients were diagnosed with medication-overuse headache. For the interictal scan, the pain specialist confirmed that patients were at least 48-h free of migraine attacks and abortive medications before the scan time. No migraine attacks were reported 3-days after the MRI scan for EM. The University of Michigan Institutional Review Board approved the study, and all participants provided written informed consent. Positron emission tomography (PET) data from the subset of the patients in the current study were previously published elsewhere [30].

**Table 1:** Demographic and clinical characteristics of study participants

characteristics	All migraine patients (*n* = 20)	EM (n = 13)	CM (n = 7)	HC (n = 26)	*P*-value^‡^
Age, years	28.5 ± 1.6	27.8 ± 1.7	29.7 ± 3.6	26.8 ± 1.4	0.428
Sex	6 M, 14 F	6 M, 7 F	7 F	7 M, 19 F	0.818
Disease duration, years	12.7 ± 1.6	12.6 ± 1.9	12.9 ± 3.1	–	NA
Frequency of migraine attack^†^	10.1 ± 1.6	5.8 ± 0.8	18.0 ± 2.0	–	NA
Aura, number	7	5	2	–	NA
HIT-6	65.3 ± 1.3	63.7 ± 1.7	68.3 ± 1.3	–	NA
Ictal thermal pain threshold (°C)	43.1 ± 1.2 (*n* = 14)	41.7 ± 1.8 (*n* = 8)	44.9 ± 1.4 (*n* = 6)	–	NA
Ictal P.A.I.N.S. (%)	13.9 ± 2.3 (*n* = 14)	14.6 ± 3.2 (*n* = 8)	12.8 ± 3.7 (*n* = 6)	–	NA
Ictal pain intensity (VAS, 0–10)	4.7 ± 0.5 (n = 14)	5.2 ± 0.4 (n = 8)	4.0 ± 1.0 (n = 6)	–	NA

^†^Average days per month. ^‡^Comparison between all migraine patients and healthy control subjects. Continuous and categorical variables were assessed by the independent two-sample *t*-test and chi-square test, respectively. The values are expressed as mean ± standard error of the mean. EM, episodic migraine; CM, chronic migraine; HC, healthy control; HIT-6, 6-item Headache Impact Test; P.A.I.N.S., Pain area and intensity number summation; M, male; F, female

### Clinical assessments

Migraine-specific variables including disease duration, presence of aura, frequency of migraine attacks per month, and the six-item Headache Impact Test (HIT-6) [31], which measures the adverse headache impact on a patient’s daily life were registered. We also used an in-house developed mobile application called PainTrek (currently named GeoPain) (MoxyTech Inc., MI) to measure sensory-discriminative pain of the ongoing migraine attacks in the craniofacial region, which is quantifiable, and validated [32]. Pain area (220 cells) and intensity number (mild pain-1, light red; moderate pain-2, red; severe pain-3, dark red) summation (P.A.I.N.S.) cumulative score (0–660) in the head and facial areas was obtained for each participant during the ictal phase and then converted to a percentage [30]. We also measured headache-related pain intensity on the standard visual analog scale (VAS) (0–10). In a previous PET study, we conducted a sustained thermal pain threshold challenge during the ictal period [30]. Migraine patients received thermal pain stimulation on the forehead trigeminal ophthalmic region, ipsilateral to the headache using a 16 × 16 mm thermode (PATHWAY system, Medoc Advanced Medical Systems, Ramat Yishai, Israel). The temperature increased 1 °C/s every 10 s starting from a 32 °C baseline to a 50 °C maximum borderline. Participants were instructed to press the mouse button when they perceived the thermal stimuli as being painful. The individual thermal pain threshold was included as an indicator of pain sensitivity (allodynia) during migraine attacks for the migraine group.

### Interictal resting-state fMRI acquisition

All MRI data were collected from a 3 T GE scanner (General Electric Medical Systems, Milwaukee, WI, USA) at the University of Michigan. fMRI data were acquired using a reverse spiral sequence [33]: repetition time = 2000 ms; echo time = 30 ms; flip angle = 90°; field of view (FOV) = 22 cm; slice thickness = 3.0 mm; Total number of volumes was 240 for the resting-state scan. T1-weighted brain image was acquired using SPGR sequence with the following parameters: repetition time = 12.22 ms; echo time = 5.176 ms; flip angle = 15°; FOV = 26 cm; number of excitations = 1; slice thickness = 1.0 mm.

Resting-state fMRI scan for migraine patients was performed during the interictal phase. During the resting-state fMRI scan, participants were instructed to keep their eyes centered on a visual fixation cross of the screen and try not to think of anything in particular, and relax. They were also asked to keep their head as still as possible during the scan. Head motion was minimized using foam pads placed around the head. The pulse oximeter was placed on the subject’s finger, and the pressure belt was placed around the abdomen of each subject so that the cardiac and respiratory signals were acquired simultaneously.

### Preprocessing and BOLD signal variability analysis

Resting-state fMRI data were reconstructed using field map correction and then corrected for cardiac- and respiratory-related noise [34]. The following preprocessing steps adapted from the 1000 Functional Connectomes Project (http://www.nitrc.org/projects/fcon_1000) [35] were conducted with the FMRIB Software Library (http://www.fmrib.ox.ac.uk/fsl) and Analysis of Functional NeuroImages (http://afni.nimh.nih.gov/afni). After discarding the first five volumes, slice time correction, motion correction, grand-mean scaling of the voxel value, removing of eight nuisance signals (cerebrospinal fluid, white matter, and six motion parameters) by regression, removing linear and quadratic trends, spatial smoothing using a Gaussian kernel of 6 mm full-width half-maximum, and temporal band-pass filtering (0.01–0.198 Hz; slow-5, 0.01–0.027 Hz; slow-4, 0.027–0.073 Hz; slow-3, 0.073–0.198 Hz) [36] were performed. The preprocessed functional images were then transformed to the Montreal Neurological Institute (MNI) (2 × 2 × 2 mm^3^) standard space using FMRIB’s Linear Image Registration Tool.

First, we calculated the BOLD_SV_ across low frequencies (0.01–0.198 Hz) and assessed group differences. It has been suggested that brain oscillation at an independent frequency band has specific properties and physiological functions [36]. Thus, we performed sub-band analysis (slow-5, 0.01–0.027 Hz; slow-4, 0.027–0.073 Hz; slow-3, 0.073–0.198 Hz) [36] to determine potential contributions of distinct frequency bands to observed overall low-frequency (0.01–0.198 Hz) differences. The standard deviation of the BOLD signal fluctuations represents the temporal variability of BOLD time-courses. The standard deviation of preprocessed functional images in standard space was calculated in each brain voxel. Each subject’s BOLD_SV_ map was standardized into a subject-level *Z*-score map by subtracting the mean of BOLD_SV_ across the whole-brain (gray matter) and then divided by the standard deviation of the BOLD_SV_ across the whole-brain (gray matter) [17]. A positive value indicates that BOLD_SV_ is higher than the whole-brain, while a negative value indicated that BOLD_SV_ is lower than the whole-brain. Voxel-wise group comparison was performed using an unpaired two-sample *t*-test. To ensure that head motion artifacts did not influence our results, we calculated the frame-wise displacement (FD) [37] for each subject. Although there was no significant group difference in mean FD (± standard deviation) (migraine patients: 0.05 ± 0.02; HC: 0.06 ± 0.03, *t* = − 1.391, *p* = 0.171); mean FD, as well as age and sex, were included as covariates in the statistical analysis to limit the potential effect of head micromovements on the BOLD_SV_ measures [38]. All results were corrected for multiple comparisons to a significance level of *p* < 0.05 (uncorrected height threshold of *p* < 0.001 [39] combined with a family-wise error (FWE)-corrected cluster-extent threshold of *p* < 0.05). Initial cluster-extent based correction (*p* < 0.05, FWE-corrected) applied in the whole-brain comparisons did not capture a significant difference in the spinal trigeminal nucleus (SpV) due to its anatomically small size. To test our hypothesis of difference in BOLD_SV_ in the SpV, we created an SpV mask with a 5-mm sphere region-of-interest (ROI) in the left (MNI *x*, *y*, *z*: − 6, − 40, − 50) and right (*x*, *y*, *z*: 6, − 40, − 50) SpV subnucleus caudalis and interpolaris [40]. Separate voxel-wise nonparametric permutation tests (two-tailed, 5000 permutations) [41] were performed on the SpV mask. The significance of group differences in the SpV was determined using the threshold-free cluster enhancement (TFCE) (*p* < 0.05, FWE-corrected for multiple comparisons), which detects clusters of contiguous voxels without having to define an initial cluster-forming height threshold. The BOLD_SV_ map of each subject was used as input data; and age, sex, and mean FD were included as covariates.

After identifying the brain regions showing significant differences between migraine and HC, we further performed subgroup analysis (EM vs. HC, CM vs. HC, and EM vs. CM). The 20 migraine patients were divided into EM (*n* = 13) or CM (*n* = 7) group. This comparison will address whether the observed BOLD_SV_ alterations are valid for both migraine groups or only for a specific subgroup.

### Cross-correlation and dynamic functional connectivity analysis

Among the significant regions identified in the between-group comparison of the BOLD_SV_, 5 ROIs in the trigeminal somatosensory pathway and 2 ROIs in frontoparietal brain regions were used for a follow-up analysis. A 5-mm radius spherical seed was generated on the peak location of significant clusters. Five ROIs in the trigeminal somatosensory pathway were defined including the right SpV (*x*, *y*, *z*: 8, − 36, − 52), left thalamus (medial pulvinar, PuM) (*x*, *y*, *z*: − 12, − 30, 10), left thalamus (ventral posteromedial, VPM) (*x*, *y*, *z*: − 12, − 20, − 2), left dorsal posterior insula (dpINS) (*x*, *y*, *z*: − 38, − 24, 16), and left primary somatosensory cortex (S1) (*x*, *y*, *z*: − 46, − 30, 60). In the current results, the left thalamic cluster includes both PuM and VPM; thus, we generated 2 peak seeds including the PuM and VPM based the Morel’s 3D thalamus segmentation [42]. Regarding the laterality of the thalamus and SpV, we selected the left thalamus (ipsilateral to the left S1/dpINS) and the right SpV (contralateral to the left thalamus) in an effort to evaluate the ascending trigeminal somatosensory pathway. Two ROIs in frontoparietal brain regions were defined including the right dorsolateral prefrontal cortex (dlPFC) (*x*, *y*, *z*: 44, 22, 36), and inferior parietal cortex (IPC) (*x*, *y*, *z*: 38, − 54, 44). The ROIs were linearly transformed to each subject’s functional space, and the mean time series (0.01–0.198 Hz) across all voxels in the ROIs were extracted. Cross-correlation analysis was performed to test whether the BOLD signal fluctuation was temporally synchronized within the pathways showing higher or lower BOLD_SV_.

To measure the dynamic changes in functional connectivity between the same ROIs, we applied the DCC (https://github.com/canlab/Lindquist_Dynamic_Correlation) [28], which is formulated in the framework of the multivariate generalized autoregressive conditional heteroscedasticity model [43]. Compared to the traditional sliding window method, the model-based DCC method was less susceptible to noise-induced temporal variability in correlations [28]. Also, DCC-derived variances of dynamic correlation were significantly more reliable than the sliding window method [44]. However, the model-based DCC method is computationally intensive. The time series (0.01–0.198 Hz) for each ROI was pre-whitened with an autoregressive moving-average (1, 1) model. Generalized autoregressive conditional heteroskedasticity models are fit to each time series to estimate conditional standard deviation. The residuals of the time series were standardized by the conditional standard deviation. An exponential weighted moving average method is applied to the standardized residuals to compute time-varying correlations (DCC). The strength and variability of dFC were quantified as the mean and standard deviation of the DCC over time [26, 45]. The strength and variability of dFC were compared between groups using an unpaired two-sample *t*-test. Statistical significance was set at *p* < 0.05.

### Clinical significance of the BOLD_SV_

The relationship between BOLD_SV_ and migraine headache severity, including P.A.I.N.S., pain VAS, and thermal pain threshold during the ictal phase, was assessed using Spearman correlation. The cross-correlation results revealed that BOLD signal fluctuation of the dlPFC and IPC were highly correlated, which indicate significant functional connectivity. Also, the location of the 2 clusters was overlapped with the right frontoparietal control network [46]. We assumed that coherent spontaneous fluctuations occur in these two regions functioning as a frontoparietal network. Thus, the extracted mean BOLD_SV_ (Z) of the right dlPFC and IPC was averaged. The Benjamini-Hochberg false discovery rate correction (*q* = 0.05) was applied for correcting multiple comparisons [47].

## Results

Demographic and clinical characteristics of the study participants are shown in Table 1. The unpaired *t*-test and chi-square test showed no significant difference between groups (migraine patients vs. HC) based on age (*p* = 0.428) and sex (*p* = 0.818). There was no significant difference between CM vs. EM patients for disease duration (*p* = 0.945), HIT-6 (*p* = 0.086), ictal thermal pain threshold (*p* = 0.202), ictal P.A.I.N.S. (*p* = 0.720), and ictal pain intensity (*p* = 0.246).

### BOLD_SV_

**Fig. 1:**
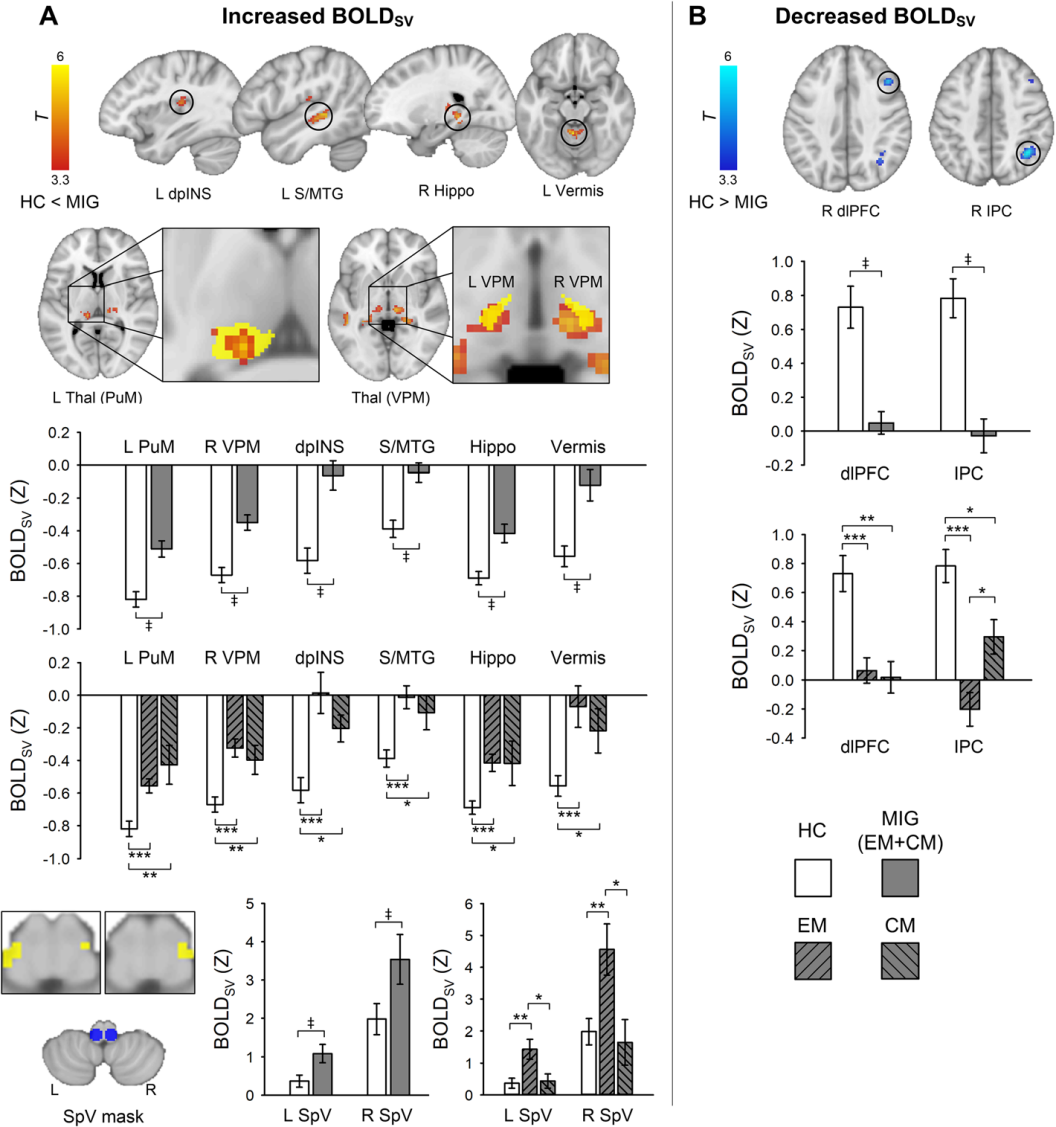
Group differences of resting-state BOLD signal variability (BOLD_SV_) within the frequency of 0.01–0.198 Hz. **a** Brain regions displaying increased BOLD_SV_ in MIG patients compared with HCs. Significant thalamic clusters are overlaid on the left PuM, VPM, and right VPM (yellow mask) of the Morel’s histology-based atlas for visualization purpose (middle). The left thalamic cluster includes both PuM and VPM. Separate voxel-wise permutation tests were performed for the SpV mask (blue), which was created with 5-mm sphere ROI in the left (*x*, *y*, *z*: − 6, − 40, − 50) and right (*x*, *y*, *z*: 6, − 40, − 50) SpV [40]. Significant clusters (yellow) were identified using a threshold-free cluster enhancement (*p* < 0.05, FWE-corrected) (bottom). **b** Brain regions displaying decreased BOLD_SV_ in MIG patients compared with HC. All statistical images are displayed with significant clusters (voxel-level threshold *p* < 0.001 and cluster-level extent threshold *p* < 0.05, FWE-corrected). Bar graphs were expressed as mean ± standard error of the mean. Mean BOLD_SV_ (Z) was extracted from a 3-mm sphere around the peak voxel of each significant cluster. Gray squares/bars represent MIG patients (*n* = 20), white squares/bars represent HC subjects (*n* = 26). MIG patients were divided into EM (*n* = 13) or CM (*n* = 7) group for further comparison. ^‡^*p* < 0.05 (FWE-corrected). ^*^p < 0.05, ^**^p < 0.01, and ^***^p < 0.001 for unpaired *t*-test. dpINS, dorsal posterior insula; S/MTG, superior and middle temporal gyrus; SpV, spinal trigeminal nucleus; Thal, thalamus; VPM, ventral posteromedial nucleus; PuM, medial pulvinar nucleus; Hippo, hippocampus; dlPFC, dorsolateral prefrontal cortex; IPC, inferior parietal cortex; L, left; R, right; MIG, migraine; HC, healthy control; EM, episodic migraine; CM, chronic migraine

We found that patients with migraine exhibited greater BOLD_SV_ in the left thalamus encompassing the PuM and VPM, right thalamus (VPM), left dpINS, superior/middle temporal gyrus, right hippocampus, and left cerebellar vermis compared with HC subjects within the entire frequency range from 0.01 to 0.198 Hz (*p* < 0.05, FWE-corrected) (Fig. 1A**,** Table 2). The peak voxels of the left and right thalamic clusters were located in the PuM and VPM, respectively, confirmed by Morel’s 3D histological atlas reconstructed in MNI space [42, 48]. The left PuM cluster extended over the left VPM. Separate voxel-wise permutation tests with TFCE in the SpV mask revealed that migraine patients displayed significantly greater BOLD_SV_ in the bilateral SpV compared with HC subjects (*p* < 0.05, FWE-corrected) (Fig. 1a**, bottom**). Conversely, patients with migraine exhibited lower BOLD_SV_ in the right dlPFC and IPC compared with HC subjects (Fig. 1b). In subgroup analyses, most of the regions were significant while comparing EM vs. HC and CM vs. HC, meaning that initial results were not changed when stratifying the patient group by headache frequency. However, greater BOLD_SV_ in the bilateral SpV was only significant in EM compared to HC, while CM patients showed a lower BOLD_SV_ in the SpV compared to EM.

**Table 2:** Brain regions with increased and decreased BOLD signal variability in migraine (EM + CM) patients compared with healthy controls

Frequency band	Contrast	Brain region	Peak MNI coordinates			Number of voxels	T score
			x	y	z		
Overall low frequency (0.01–0.198 Hz)	Migraine > Controls	Left spinal trigeminal nucleus	−10	−38	−48	4	3.73*
		Right spinal trigeminal nucleus	8	−36	−52	7	3.93*
		Left thalamus	−12	−30	10	166	5.14
		Right thalamus	10	−22	−2	298	5.11
		Left dorsal posterior insula	−38	−24	16	114	5.01
		Left middle temporal gyrus	−48	−36	0	75	5.31
		Right hippocampus	22	−36	−2	69	4.87
		Left cerebellar vermis	−2	−48	−16	127	5.96
	Migraine < Controls	Right dorsolateral prefrontal cortex	44	22	36	75	5.41
		Right inferior parietal cortex	38	−54	44	205	5.99
Slow-5 (0.01–0.027 Hz)	Migraine > Controls	Left thalamus	−12	−30	6	78	4.99
	Migraine < Controls	Right inferior parietal cortex	38	−54	44	129	5.50
Slow-4 (0.027–0.073 Hz)	Migraine > Controls	Left thalamus	−12	−30	10	109	4.67
		Right thalamus	10	−22	−2	107	4.46
		Left dorsal posterior insula	−34	−22	16	123	4.53
		Left primary somatosensory cortex	−46	−30	60	56	4.46
	Migraine < Controls	Right dorsolateral prefrontal cortex	44	22	36	61	4.76
		Right inferior parietal cortex	40	−54	44	78	4.61
		Left angular gyrus	−50	− 62	20	58	4.19
Slow-3 (0.073–0.198 Hz)	Migraine > Controls	Left spinal trigeminal nucleus	−8	−36	−48	11	3.60*
		Right spinal trigeminal nucleus	8	−36	−52	16	3.86*
		Left thalamus	−14	−10	4	263	4.87
		Right thalamus	22	−24	12	146	5.80
		Right dorsal posterior insula	36	−24	22	58	4.86
		Left middle temporal gyrus	−48	−38	0	84	4.57
		Right hippocampus	26	−38	−8	125	5.34
		Left cerebellar vermis	−4	−48	−18	345	6.1
	Migraine < Controls	Right dorsolateral prefrontal cortex	44	24	40	88	5.31
		Right inferior parietal cortex	38	− 56	44	273	6.61
		Right angular gyrus	50	−60	16	86	5.26
		Left inferior parietal cortex	−40	−56	46	102	4.86

All statistical results were thresholded at voxel-level *p* < 0.001 and cluster-level *p* < .05, FWE-corrected. *p < .05, FWE-corrected using TFCE in the spinal trigeminal nucleus mask

**Fig. 2:**
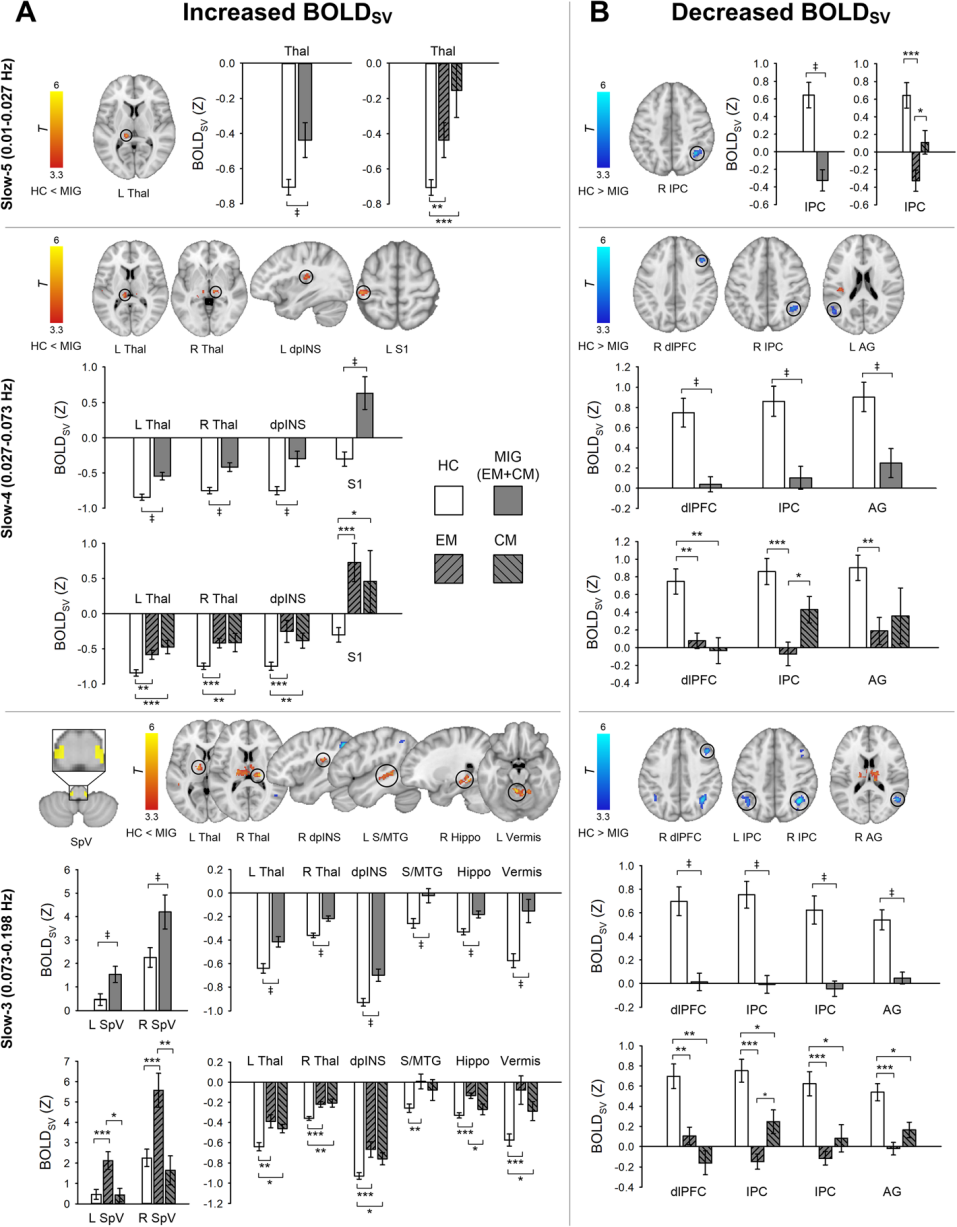
Group differences of BOLD_SV_ in the frequency band slow-5 (0.01–0.027 Hz), slow-4 (0.027–0.073 Hz), and slow-3 (0.073–0.198 Hz). **a** Brain regions displaying increased BOLD_SV_ in MIG patients compared with HCs. Separate voxel-wise permutation tests in each frequency band were performed for the SpV mask. Significant clusters (yellow) were identified using a threshold-free cluster enhancement (*p* < 0.05, FWE-corrected) (bottom). **b** Brain regions displaying decreased BOLD_SV_ in MIG patients compared with HCs. All statistical images are displayed with significant clusters (voxel-level threshold *p* < 0.001 and cluster-level extent threshold *p* < 0.05, FWE-corrected). Bar graphs were expressed as mean ± standard error of the mean. Mean BOLD_SV_ (Z) was extracted from a 3-mm sphere around the peak voxel of each significant cluster. Gray squares/bars represent MIG patients (n = 20), white squares/bars represent HC subjects (n = 26). MIG patients were divided into EM (n = 13) or CM (n = 7) groups for further subgroup comparison. ^‡^*p* < 0.05 (FWE-corrected). ^*^p < 0.05, ^**^p < 0.01, and ^***^p < 0.001 for unpaired *t*-test. S1, primary somatosensory cortex; AG, angular gyrus; MIG, migraine; HC, healthy control; EM, episodic migraine; CM, chronic migraine

To further verify frequency-specific perturbation in BOLD_SV_, frequency range was divided into slow-5 (0.01–0.027 Hz), slow-4 (0.027–0.073 Hz), and slow-3 (0.073–0.198 Hz). Within the slow-5 frequency band (Fig. 2**, top**), migraine patients had greater BOLD_SV_ in the left thalamus corresponding to the PuM but showed lower BOLD_SV_ in the right IPC compared with HCs. In subgroup analyses, the CM group showed marginally lower BOLD_SV_ in the right IPC (*p* = 0.07) compared with HCs. Within the slow-4 frequency band (Fig. 2**, middle**), we observed greater BOLD_SV_ in the left PuM, dpINS, S1, and right thalamus encompassing VPM, and ventral posterolateral nucleus in the migraine patients compared with HCs. In contrast, BOLD_SV_ in the right dlPFC, IPC, and left angular gyrus was significantly lower in migraine patients. In subgroup analyses, initial results remained similar except in some regions. BOLD_SV_ in the right IPC (*p* = 0.16) and left angular gyrus (*p* = 0.10) was not significant between CM vs. HC. Within the slow-3 frequency band (Fig. 2**, bottom**), we found greater BOLD_SV_ in the right dpINS, hippocampus, left superior/middle temporal gyrus, cerebellar vermis, left thalamus encompassing medial dorsal nucleus, thalamic reticular nucleus, and ventral lateral nucleus; and the right thalamus encompassing the ventral posterolateral, lateral posterior nucleus, VPM, and PuM. Additional voxel-wise permutation tests with TFCE in the SpV mask confirmed that migraine patients displayed significantly greater BOLD_SV_ in the bilateral SpV compared with HC subjects (*p* < 0.05, FWE-corrected). Similar to other frequency bands, BOLD_SV_ in the right dlPFC, angular gyrus, and bilateral IPC was significantly lower in migraine patients compared with HCs. In subgroup analysis comparing EM vs. HC and CM vs. HC, most of the regions were significant. However, greater BOLD_SV_ in the bilateral SpV, left superior/middle temporal gyrus, and right hippocampus was only significant in EM compared to HC. The CM group showed lower BOLD_SV_ in the bilateral SpV and right hippocampus compared to EM. Also, the CM group showed marginally lower BOLD_SV_ in the right dlPFC (*p* = 0.08) compared with EM.

### Cross-correlation in the trigeminal spinal-thalamo-cortical pathway and frontoparietal pathway

**Fig. 3:**
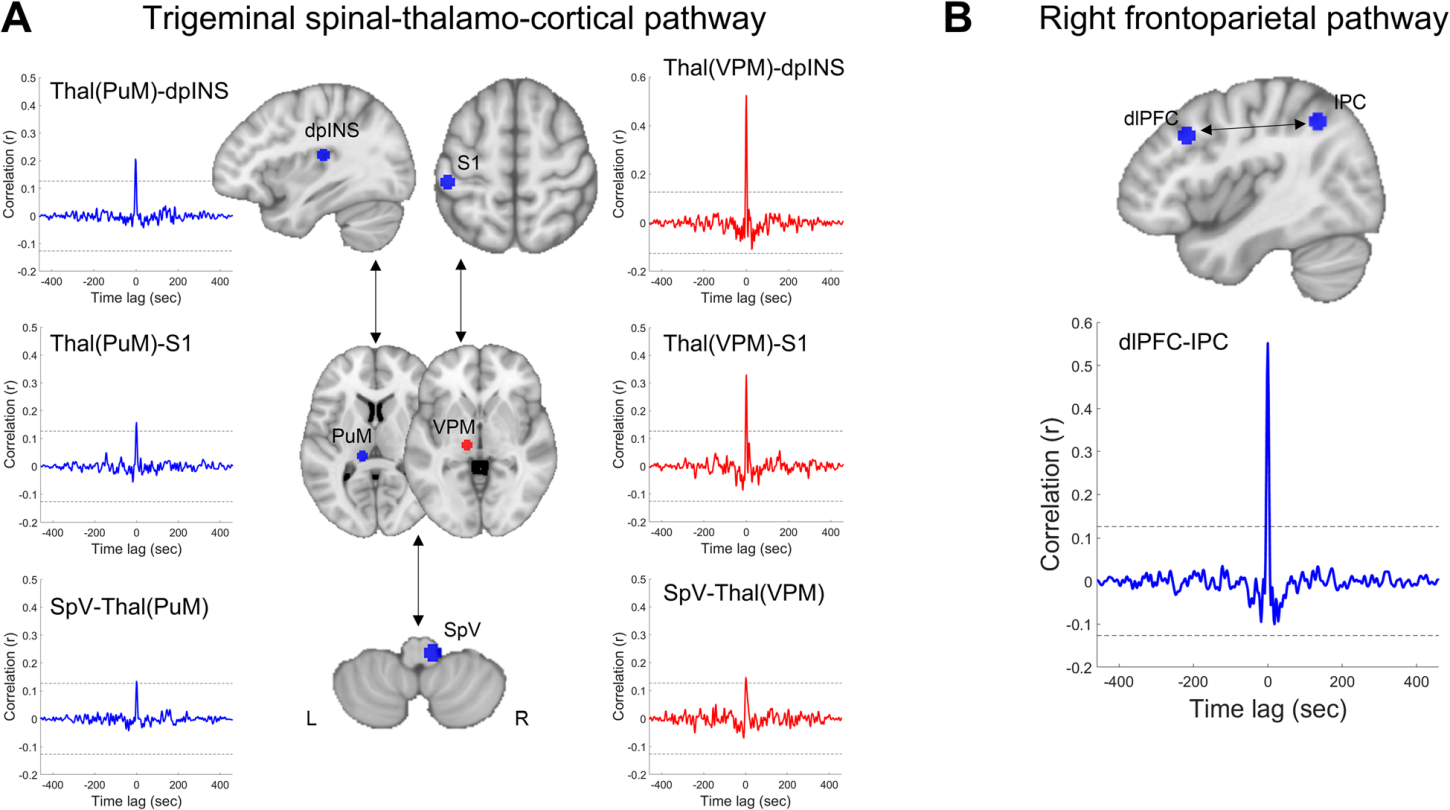
Cross-correlation of the resting-state BOLD signal time course. Cross-correlation of the BOLD signal within the trigeminal spinal-thalamo-cortical pathway (**a**), which was increased BOLD_SV_ and right frontoparietal region (**b**), which was decreased BOLD_SV_. Blue and red masks in the brain image indicate the locations of the maximum difference of BOLD_SV_ within the frequency of 0.01–0.198 Hz except for S1, which was significant in the slow-4 (0.027–0.073 Hz) band. All time courses (0.01–0.198 Hz) were extracted from masks (5-mm sphere) to calculate cross-correlation between the regions. The blue and red lines indicate grand averaged cross-correlation across all 46 subjects. The upper and lower horizontal dotted lines indicate the approximate 95% confidence bounds

Grand averaged (*n* = 46) cross-correlation (0.01–0.198 Hz) graph showed a significant correlation between pairs of regions. The results indicated that the BOLD signal fluctuation was temporally synchronized within the trigeminal spinal-thalamo-cortical pathway (Fig. 3a), and within the frontoparietal pathway (Fig. 3b).

### Dynamic functional connectivity

**Fig. 4:**
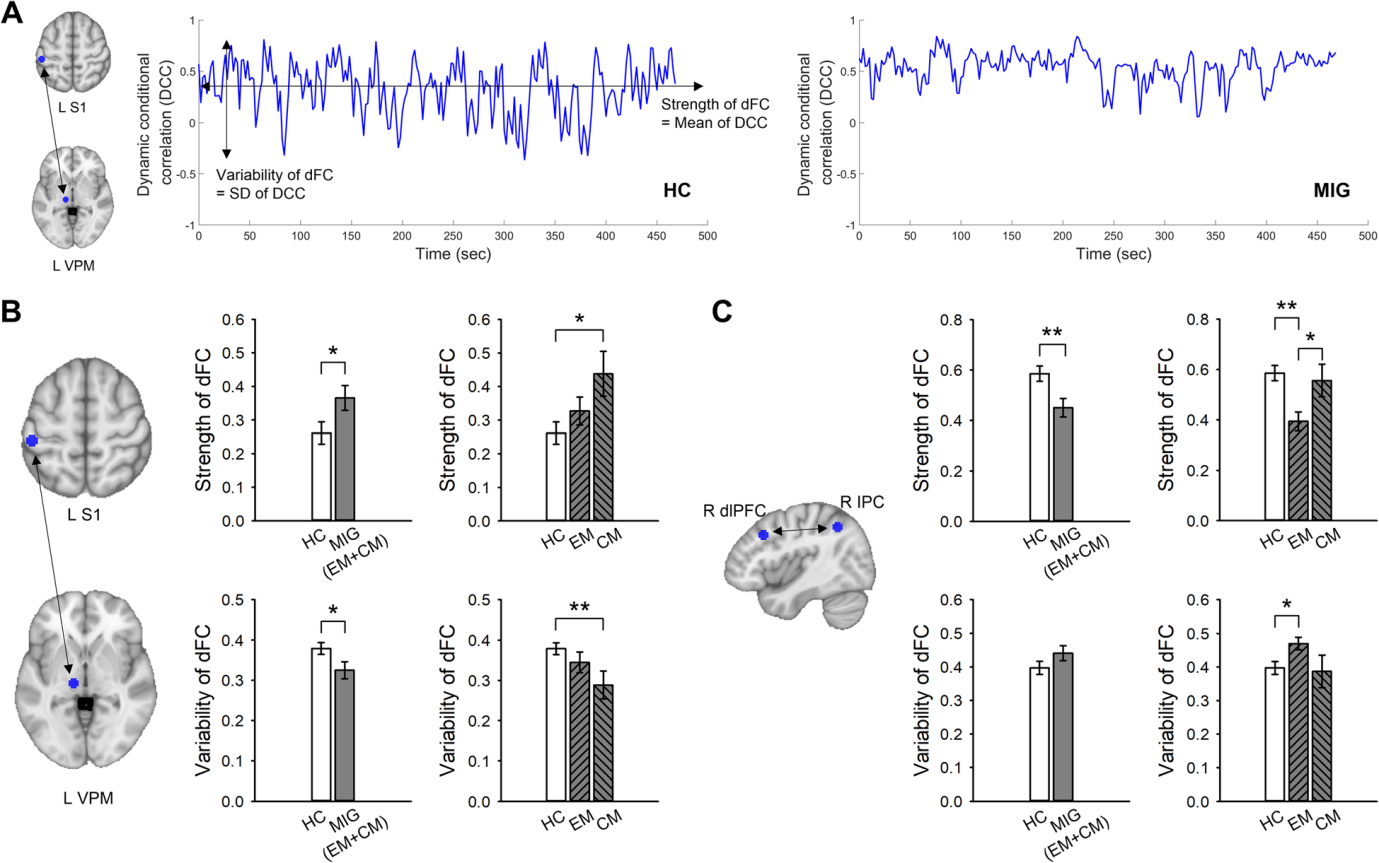
Example of dynamic conditional correlation between the ventral posteromedial thalamic nucleus (VPM) and primary somatosensory cortex (S1), which have been filtered at 0.01–0.198 Hz (**a**) and group differences of dynamic functional connectivity (dFC) in the thalamocortical (**b**) and frontoparietal pathways (**c**). The strength and variability of dFC were derived by the mean and standard deviation of the DCC over time, respectively. Bar graphs of dFC strength and variability were expressed as mean ± SEM. Gray squares/bars represent MIG patients (n = 20) and white squares/bars represent HC subjects (n = 26). MIG patients were divided into EM (n = 13) or CM (n = 7) group for further subgroup comparison. ^*^p < 0.05 and ^**^p < 0.01 for unpaired *t*-test. L, left; R, right; dlPFC, dorsolateral prefrontal cortex; IPC, inferior parietal cortex; HC, healthy control; MIG, migraine; EM, episodic migraine; CM, chronic migraine

We then applied DCC analysis to characterize temporal dynamics of functional connectivity. The left and right panel of Fig. 4a shows a time series of dFC (0.01–0.198 Hz) between the pair of BOLD signal (VPM-S1) using the DCC model in representative HC and MIG subjects, respectively. We found significant group differences in both strength (*p* < 0.05) and variability (*p* < 0.05) of dFC between VPM and S1 (Fig. 4b). The strength of dFC (VPM-S1) was significantly greater (*p* < 0.05) in CM patients compared with HC subjects, while the variability of dFC (VPM-S1) was lower (*p* < 0.01) in CM patients compared with HC subjects. There were no significant group differences in the dFC between the SpV and VPM, and between the VPM and insula (all *p* > 0.05). In the frontoparietal pathway, the strength of dFC between right dlPFC and IPC was decreased in migraine patients compared with HC (*p* < 0.01) (Fig. 4c). This difference was mainly driven by EM patients. EM patients showed a reduction in dFC strength compared to the HC (*p* < 0.01) and CM (*p* < 0.05) groups. In addition, we found higher variability of dFC in EM patients compared with the HC group (*p* < 0.05).

### Correlation with migraine headache severity

**Fig. 5:**
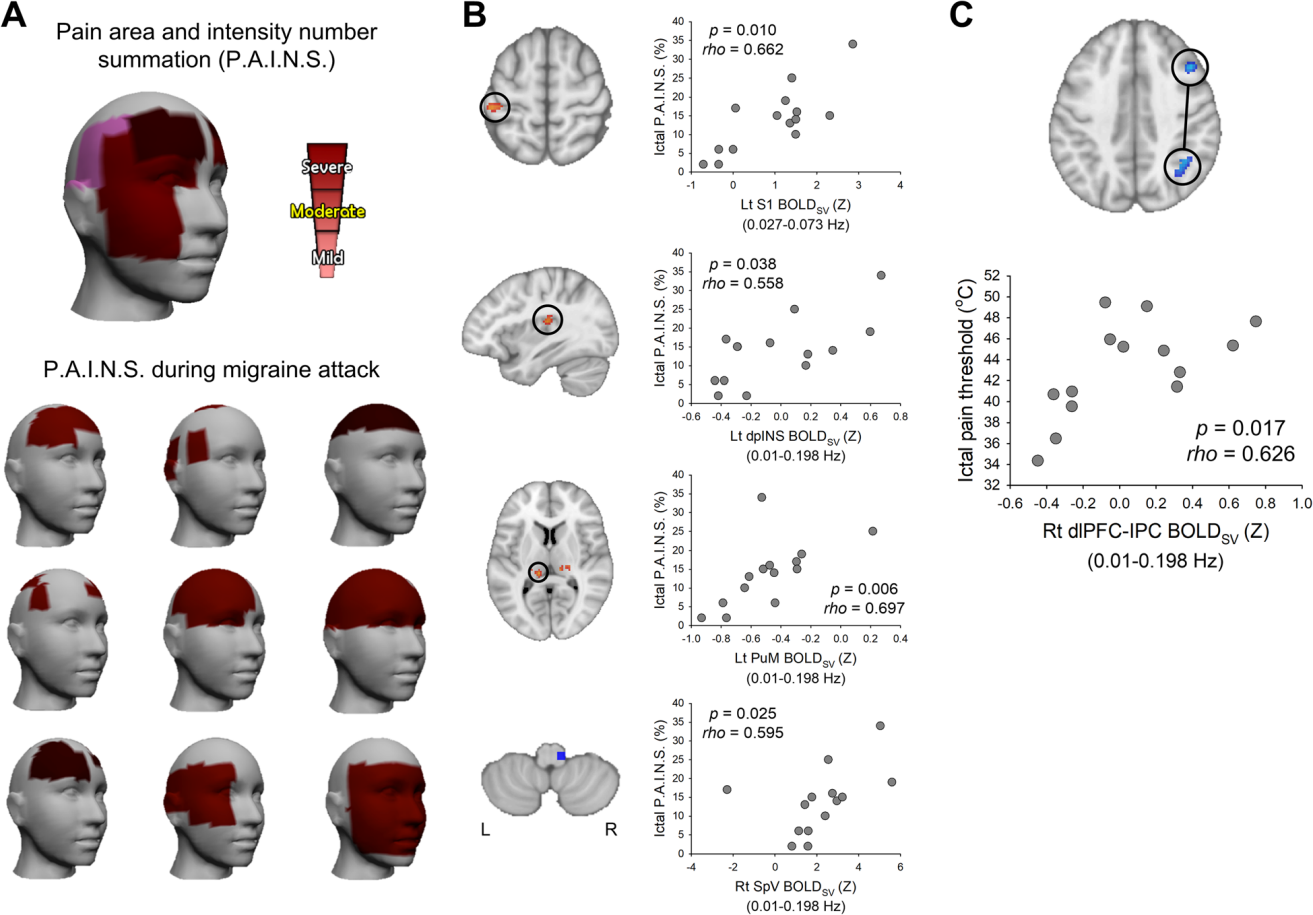
Correlation between BOLD signal variability (BOLD_SV_) and migraine headache severity. **a** Migraine headache severity was assessed by using mobile application PainTrek (currently named GeoPain) (MoxyTech Inc., MI) during migraine attacks. Pain area (220 cells) and intensity number (mild pain-1, light red; moderate pain-2, red; severe pain-3, dark red) summation cumulative score (0–660) was converted to a percentage. Example 3D head image shows individually recorded P.A.I.N.S. from migraine patients during headache attacks. **b** The black circles in the brain image indicate the locations of the maximal difference (control < migraine) in BOLD_SV_ within the frequency of 0.01–0.198 Hz except for S1, which was significant in the slow-4 (0.027–0.073 Hz) band. Mean BOLD_SV_ (Z) was extracted from a 3-mm sphere around the peak voxel of each cluster. **c** The thermal pain threshold during migraine attacks was measured on the ophthalmic trigeminal region. The black circles in the brain image indicate the locations of the maximal difference (control > migraine) in BOLD signal variability within the frequency of 0.01–0.198 Hz. Extracted mean BOLD_SV_ (Z) of the right dlPFC and IPC was averaged

Figure 5a shows individually measured P.A.I.N.S. in 9 representative patients during their migraine attacks. Figure 5b shows the relationship between BOLD_SV_ in the ascending trigeminal somatosensory pathway and migraine headache severity in the patient group. Patients with higher BOLD_SV_ in the right SpV (*rho* = 0.595, *p* = 0.025, *q* = 0.0417), left PuM (*rho* = 0.697, *p* = 0.006, *q* = 0.025), dpINS (*rho* = 0.558, *p* = 0.038, *q* = 0.047), and S1 (*rho* = 0.662, *p* = 0.010, *q* = 0.025) had greater P.A.I.N.S. in the head and facial area (Fig. 5b). No statistically significant relationship was found between right VPM variability and P.A.I.N.S. (*rho* = 0.406, *p* = 0.150, *q* = 0.150). Also, correlation between headache pain intensity (VAS) and BOLD_SV_ in the right SpV (*rho* = 0.587, *p* = 0.027, *q* = 0.067), left PuM (*rho* = 0.506, *p* = 0.065, *q* = 0.081), dpINS (*rho* = 0.513, *p* = 0.061, *q* = 0.081), and S1 (*rho* = 0.455, *p* = 0.102, *q* = 0.102) were not significant after false discovery rate correction. In the frontoparietal pathway, patients with lower BOLD_SV_ in the dlPFC and IPC had lower thermal pain threshold on the ophthalmic trigeminal region during migraine attacks (*rho* = 0.626, *p* = 0.017) (Fig. 5c). There were no other significant correlations.

## Discussion

Our results revealed that migraineurs displayed significantly greater BOLD_SV_ in the SpV, PuM, VPM, S1, and dpINS, which constitute the ascending trigeminal somatosensory pathway, in addition to the auditory association cortex, hippocampus, and cerebellar vermis. Conversely, the patients exhibited lower BOLD_SV_ in the top-down pain modulatory circuits, including the dlPFC and IPC. The subgroup analysis confirmed that both EM and CM groups have similar abnormalities of BOLD_SV_ in most of the significant regions. By applying DCC analysis, we found that migraineurs exhibited less variability and greater strength of dFC in the thalamo-cortical pathway (VPM-S1) than HCs. In contrast, migraine patients showed higher variability and lower strength of dFC in the frontoparietal pathway (dlPFC-IPC). Finally, we demonstrated that dysfunctional interictal BOLD_SV_ in the ascending trigeminal somatosensory pathway and frontoparietal pathways were correlated with the patient’s headache severity and thermal pain sensitivity during migraine attacks.

It is noteworthy that migraineurs exhibited greater temporal variability of BOLD signal in the trigeminal somatosensory pathway, which is involved in the core pathophysiology of migraine [1]. For example, the SpV in the brainstem, which receives cranial and orofacial noxious afferents, has been suggested to be involved in the generation of migraine attacks [49, 50]. The posterior thalamus plays a vital role in widespread allodynia during a migraine attack [51]. At the cortical level, increased S1 excitability in the interictal state [52] and cortical thickness changes in the S1 [53, 54] were reported. The dpINS, having a crucial role in pain perception [55], was reported as a promising target region for migraine treatment [56].

It has been suggested that greater signal variability was known to reflect a greater range of neuronal responses, which is achieved by balanced synaptic excitation and inhibition, for better adaptive function in a given environment [13, 57]. However, higher brain signal variability compared to HC subject, presented in our migraine patients, can be interpreted as pathological. Abnormally elevated dynamic range in the trigeminal spinal-thalamo-cortical pathway may amplify nociceptive and sensory information processing during and between attacks. The positive correlation between BOLD_SV_ in this pathway and individuals’ P.A.I.N.S. during migraine attacks supports this interpretation. Taken together, increased resting-state brain signal instability in the trigeminal somatosensory pathway may contribute to an abnormal pain and sensory gain during and even outside of migraine attacks [2].

Our findings of abnormally increased BOLD_SV_ in the trigeminal somatosensory pathway and altered thalamo-cortical dFC are in agreement with thalamo-cortical dysrhythmia in interictal migraine [8, 27, 58–61] and other chronic pain conditions [40, 62, 63]. Prior electrophysiological study in migraine found evidence of abnormalities in somatosensory [61] or visual [59] evoked high-frequency oscillations, which is suggestive of diminished thalamo-cortical activity. Moreover, impaired lateral inhibition of somatosensory evoked potentials in migraine between attacks, likely due to insufficient thalamocortical drive, was correlated with the intensity and duration of migraine attacks [64]. A recent study in CM patients has shown that the degree of lateral inhibition of somatosensory evoked potentials was associated with attack frequency [65]. These reduced interictal thalamo-cortical drives in migraines may result from low brainstem activation [66]. Together, abnormal thalamic variability and thalamo-cortical dynamic interactions may lead to deficient habituation to sensory stimuli in migraine [67].

In previous resting-state fMRI studies in migraine, increased amplitude of low-frequency oscillation was found in the medial dorsal nucleus of the thalamus in slow-4 (0.027–0.073 Hz) band [8]. This result is in line with our findings of aberrant signal variability in the higher-order relays of the thalamus in migraine. Herein, one of the primary sources of increased BOLD_SV_ within the frequency range from slow-5 to slow-4 was located in the left PuM. The medial pulvinar is the higher-order relay nucleus of the thalamus. It has a reciprocal connection with the higher-order cortex and paralimbic areas, including prefrontal, posterior parietal, insula, and parahippocampal cortices [68]. Thus, abnormal signal variability in the PuM would result in disrupted thalamo-cortical information flow and, in turn, lead to alteration of multisensory integration and higher cognitive processing.

Importantly, migraine patients displayed less variability of dFC accompanied with the increased strength of dFC between VPM and S1 compared with the HC group. These abnormalities were prominent in the CM group. The VPM and S1 are thought to be involved in trigeminal nociceptive information processing [6]. It was proposed that high variability of dFC indicates instability of information transfer, whereas high strength of dFC indicates stable brain network integrity [45]. Therefore, altered variability and strength of dFC within the ascending trigeminal somatosensory pathway in CM patients may reflect strengthened network integration for nociceptive information processing. Consistent with this concept, a recent study has shown that abnormal posterior thalamo-cortical dynamic functional network connectivity was associated with the frequency of headache attacks in migraine [27].

We found lower BOLD_SV_ in the higher-order prefrontal and parietal association cortex in migraine patients compared to HCs. The dlPFC and posterior parietal cortex are critically involved in the pathophysiology of chronic pain, including migraine [69–72]. In our results, the BOLD signal fluctuations in the right dlPFC and IPC were highly correlated, and the significant clusters were overlapped with the frontoparietal control network playing a crucial role in endogenous pain control [73]. Both the dlPFC and IPC have been implicated in the top-down attentional control processes for pain [74–76]. It has been suggested that the temporal variability of neural activity is related to the efficiency of neural systems. Likewise, the presence of an optimal level of noise may facilitate neural function [12, 77]. Thus, lower levels of BOLD_SV_ in the dlPFC and IPC regions could result in inadequate top-down pain modulatory function. We confirmed this by demonstrating a significant correlation between decreased BOLD_SV_ in the dlPFC-IPC and lower thermal pain threshold on the ophthalmic trigeminal region measured during migraine attacks. This argument accords with the view that interictal dysfunction of the descending pain modulatory system could contribute to central sensitization during migraine attacks [9, 78].

Moreover, we found increased variability along with reduced strength of dFC between right dlPFC and IPC in EM patients. The frontoparietal network has been reported to play an important role in cognitive control and top-down modulation of pain [73]. Thus, our results indicated disruption of right frontoparietal network integrity and compromised within-network information propagation, which might contribute to impaired endogenous pain modulatory function. These findings align with the previous studies that migraineurs had altered right frontoparietal network functional connectivity (static) during the interictal period [79, 80].

It was indicated that temporal variability of resting-state BOLD signal and dynamic network connectivity would be related to trait-like (longer-lasting) pain characteristics. Using a machine learning approach, Rogachov and colleagues demonstrated that baseline BOLD_SV_ in the S1 (ascending pain pathway) and posterior cingulate cortex (default-mode network) could predict patients’ average (trait) pain [17]. Furthermore, cross-network dFC in neuropathic pain reflected a trait-like pain feature [25]. In our current study, we assessed the migraine headache area/intensity (P.A.I.N.S.) and thermal pain threshold when the patients were in the ictal period. Thus, those clinical variables could be regarded as trait-like pain. Altogether, baseline BOLD_SV_ during the interictal period can be used to predict trait-like migraine characteristics such as the severity of the migraine attack.

Some limitations need to be considered when interpreting the data. Resting-state BOLD_SV_ analysis included both EM and CM for the patient group, and thus the results may not be specific for either EM or CM. However, the subgroup analysis confirmed that both EM and CM groups have comparable abnormalities of BOLD_SV_ in most of the significant regions. Future studies are warranted using a larger sample size of migraine patients to confirm the validity and reliability of the current results and to provide more specific information regarding migraine subtype (e.g., episodic vs. chronic or aura vs. without aura). Lastly, although we intent to match the sex ratio between diagnostic groups (migraine vs. HC) and adjust potential sex effect on the BOLD_SV_ in statistical analyses, the entirety of the CM group being female may limit the applicability of the subgroup analysis and thus the results may be affected by that factor. An extension study with larger sample size and a balanced sex ratio between subgroups is warranted.

## Conclusions

Our study provides evidence of altered brain dynamics by demonstrating bi-directional changes in signal variability and time-varying connectivity within the ascending trigeminal somatosensory vs. top-down modulatory pathways in migraineurs. We demonstrated that dysfunctional interictal BOLD_SV_ in the ascending trigeminal somatosensory pathway and frontoparietal pathways were associated with the patient’s headache severity and thermal pain sensitivity during migraine attacks. Contrasting dFC patterns in the thalamo-cortical (VPM-S1) and frontoparietal (dlPFC-IPC) pathways could be linked to abnormal network integrity and instability for pain transmission and modulation.

## Data Availability

The data supporting the findings of this study are available from the corresponding author upon reasonable request.

## Abbreviations

AG: Angular gyrus
ALFF: Amplitude of low-frequency fluctuation
BOLD: Blood-oxygen-level-dependent
BOLD_SV_: Blood-oxygen-level-dependent signal variability
CM: Chronic migraine
DCC: Dynamic conditional correlation
dFC: Dynamic functional connectivity
dlPFC: Dorsolateral prefrontal cortex
dpINS: Dorsal posterior insula
EM: Episodic migraine
fMRI: Functional magnetic resonance imaging
FD: Frame-wise displacement
FOV: Field of view
FWE: Family-wise error
HC: Healthy controls
Hippo: Hippocampus
HIT-6: 6-item Headache Impact Test
IPC: Inferior parietal cortex
MIG: Migraine
MNI: Montreal Neurological Institute
MRI: Magnetic resonance imaging
P.A.I.N.S.: Pain area and intensity number summation
PET: Positron emission tomography
PuM: Medial pulvinar nucleus
ROI: Region-of-interest
S1: Primary somatosensory cortex
S/MTG: Superior and middle temporal gyrus
SpV: Spinal trigeminal nucleus
TFCE: Threshold-free cluster enhancement
VPM: Ventral posteromedial nucleus
